# A Protective Role of Canonical Wnt/*β*-Catenin Pathway in Pathogenic Bacteria-Induced Inflammatory Responses

**DOI:** 10.1155/2024/8869510

**Published:** 2024-02-27

**Authors:** Zhongjia Jiang, Weiping Zhou, Xing Tian, Peng Zou, Ning Li, Chunmeng Zhang, Yanting Li, Guangyan Liu

**Affiliations:** ^1^Department of Biochemistry and Molecular Biology, Shenyang Medical College, Shenyang 110034, China; ^2^Key Laboratory of Environment Pollution and Microecology of Liaoning Province, Shenyang 110034, China; ^3^Department of Pathogen Biology, Shenyang Medical College, Shenyang 110034, China; ^4^Department of Physiology, Shenyang Medical College, Shenyang 110034, China

## Abstract

Inflammation is a complex host defensive response against various disease-associated pathogens. A baseline extent of inflammation is supposed to be tightly associated with a sequence of immune-modulated processes, resulting in the protection of the host organism against pathogen invasion; however, as a matter of fact is that an uncontrolled inflammatory cascade is the main factor responsible for the host damage, accordingly suggesting a significant and indispensable involvement of negative feedback mechanism in modulation of inflammation. Evidence accumulated so far has supported a repressive effect of the canonical Wnt/*β*-catenin pathway on microbial-triggered inflammation via diverse mechanisms, although that consequence is dependent on the cellular context, types of stimuli, and cytokine environment. It is of particular interest and importance to comprehend the precise way in which the Wnt/*β*-catenin pathway is activated, due to its essential anti-inflammatory properties. It is assumed that an inflammatory milieu is necessary for initiating and activating this signaling, implying that Wnt activity is responsible for shielding tissues from overwhelming inflammation, thus sustaining a balanced physiological condition against bacterial infection. This review gathers the recent efforts to elucidate the mechanistic details through how Wnt/*β*-catenin signaling modulates anti-inflammatory responses in response to bacterial infection and its interactions with other inflammatory signals, which warrants further study for the development of specific interventions for the treatment of inflammatory diseases. Further clinical trials from different disease settings are required.

## 1. Inflammation

Inflammation is a complex host defensive response against various detrimental stimuli such as radiation, injury, and pathogens. Scientific research has shown the pivotal role of inflammatory components in initiating and perpetuating infectious diseases. A baseline expression of cytokines and chemokines is involved in immune-mediated processes, thus protecting host organisms against pathogen invasion. Nevertheless, emerging evidence suggests that an overwhelming inflammatory reaction is the main cause of inflammatory diseases [1, 2]. Therefore, it is conceivable that a fundamental puzzle in immunology is how the innate immune system launches robust inflammatory responses against pathogens while maintaining a considerable state of tolerance to prevent host tissues from damage. In this context, several signaling transduction pathways have been well characterized to play vital roles in the motivation and maintenance of inflammation; however, the cellular mechanisms that negatively regulate excessive inflammation are currently unresolved. Rather than being seen as a passive way of restraining pro-inflammatory responses, anti-inflammatory responses should be actively conducted and initiated simultaneously to limit the duration and strength of pro-inflammatory responses [3, 4]. Consequently, the identification of further anti-inflammatory mechanisms and the delineation of the signaling pathways involved will bring fresh perspectives on the regulation of inflammation, as well as generate additional therapeutic targets for treating diseases associated with inflammation.

## 2. Canonical Wnt Signaling Pathway

**Figure 1:**
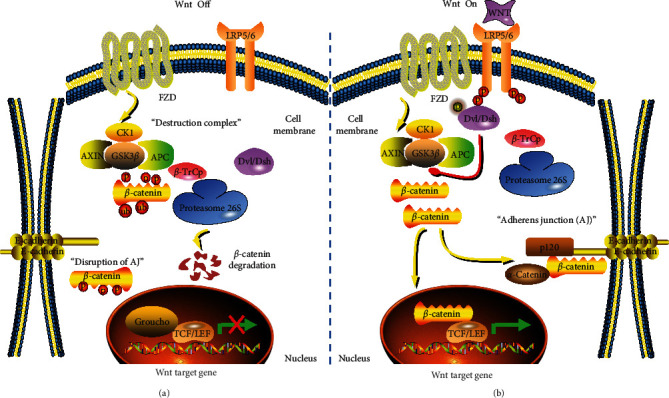
Canonical Wnt/*β*-catenin pathway in mammals. (a) Under resting conditions, the cytosolic *β*-catenin is phosphorylated by GSK3*β* in a “destruction complex” consisting of AXIN, APC, CK1, and GSK3*β*, resulting in its ubiquitination and degradation, thereby inhibiting Wnt signaling and disrupting the formation of adherens junction (AJ) by making *β*-catenin unavailable (Wnt Off). (b) Upon Wnt ligand binding to FZD and LRP5/6, an adaptor protein Dvl/Dsh is recruited and phosphorylated. Phosphorylated Dvl/Dsh then inactivates GSK3*β*, allowing cytosolic retention of *β*-catenin, from where it can interact with E-cadherin at the cell membrane to enhance cellular adhesion. The accumulated and nuclear translocation of *β*-catenin subsequently forms a complex with TCF/LEF proteins by removing transcriptional repressors (e.g., Groucho), initiating the transcription of Wnt target genes (Wnt On).

Canonical Wnt signaling contains several evolutionarily conserved cellular components and is pivotal in mediating tissue homeostasis and embryonic development [5]. It is characterized by the interaction between the Wnt ligand and particular targets, characterizes it, resulting in a regulation of *β*-catenin activity that is contingent on its protein abundance and nuclear localization. Under normal conditions, the proteolytic degradation of cytosolic *β*-catenin by a “destruction complex” comprising axis inhibition protein (AXIN), adenomatous polyposis coli (APC), casein kinase 1 (CK1), and glycogen synthase kinase 3*β* (GSK3*β*) is sufficient to maintain its minimal level. GSK3*β* targets *β*-catenin via phosphorylation of threonine41, serine33, and serine37 residues, thus allowing the F-box E3 ubiquitin ligase complex (*β*-TrCP) to bind and tag *β*-catenin for proteasome 26S-mediated degradation (Figure 1, Wnt Off) [6, 7]. The secretion of Wnt ligands, either autocrine or paracrine, contributes to the activation of signaling and enables it to attach to the frizzled (FZD) receptors as well as their corresponding low-density lipoprotein receptor-related protein 5/6 (LRP5/6) coreceptors. Formation of the ligand–receptor complex triggers binding of the *β*-catenin destruction complex, followed by the recruitment and phosphorylation of Dishevelled (Dvl/Dsh) from the cytoplasm to the plasma membrane, which then inactivates and sequesters GSK3*β* from its cytoplasmic substrates, thus leading to the disassembly of the destruction complex [8, 9]. This permits the stabilization and subsequent cytoplasmic accumulation of *β*-catenin, from where it can translocate to the nucleus. By displacing Groucho as corepressor, T cell factor/lymphoid enhancer-binding factor (TCF/LEF) proteins bind with it, thus initiating the transcription of downstream gene sets that are specific to the region in question (Figure 1, Wnt On). Additionally, the cytoplasmic *β*-catenin pool functions as a part of adherens junction (AJ) in conjunction with transmembrane protein E-cadherin, thus augmenting cellular adhesion [10, 11].

Although recent studies have characterized the critical role of Wnt signaling in the pathogenesis of chronic inflammatory diseases, its properties in modulating host immune responses during microbial infection remain poorly understood. Canonical Wnt/*β*-catenin signaling has been demonstrated to regulate inflammatory responses in various cell types depending on the context, stimuli, or crosstalk with other signaling pathways. Accordingly, it is not surprising that Wnt signaling possesses different strategies to induce or suppress immune responses in accordance with different conditions. However, accumulating evidence so far has been more supportive of the inhibitory roles of canonical Wnt signaling in the regulation of bacteria-triggered inflammation. Herein, a whole range of experimental data confirming such a notion will be clearly described.

## 3. Canonical Wnt/*β*-Catenin Pathway in Pathogen-Triggered Anti-Inflammatory Responses

As upstream mediators, Wnt family members possess significant functions in modulating host inflammation, although their role in pro- or anti-inflammatory processes is still controversial. It is generally believed that classical Wnt signals inhibit inflammation and nonclassical Wnt signals promote inflammation. Recent reports have identified the anti-inflammatory effects of classical Wnt signaling in various microbial infections. Neumann et al. [12] demonstrated that infection of murine macrophage BMDMs with *Mycobacterium tuberculosis (M. tuberculosis*) dose-dependently up-regulated FZD1 expression at the transcription level by up to 15-fold, which predominantly serves as a mediating cellular response to Wnt3a. In this case, a significant decrease in TNF expression was detected in the presence of Wnt3a CM (Wnt3a-conditional medium, a canonical Wnt signaling ligand), in part via up-regulating FZD1 and down-regulating the TLR/NF-*κ*B pathway, thus indicating an anti-inflammatory effect of Wnt3a in *M. tuberculosis*-induced inflammatory responses. Similarly, *Mycobacterium bovis* BCG infection of murine alveolar RAW264.7 macrophages, as a *Mycobacterium* model, was found to cause consistent activation of pro-inflammatory cytokines, such as TNF*α* and IL-6, whose transcriptional activities were potently attenuated when the cells were exposed to Wnt3a CM [13]. These findings were consistent with recent studies in *Pseudomonas aeruginosa* (PA)-challenged RAW264.7, in which diminished pro-inflammatory cytokines including IL-1*β*, TNF*α*, and MIP-2 (macrophage inflammatory protein 2) were observed in the presence of Wnt3a CM [14]. A noteworthy result of the Wnt3a CM addition was a marked augmentation in *Arg1* transcription, a M2-associated marker gene responsible for encoding Arginase-1 protein [15], in primary murine macrophages, suggesting that it could be driving macrophage polarization toward an M2-like phenotype during pathogenic bacteria infection and consequently resulting in a weakened immune response [12]. Such a fresh notion was also supported by a recent study using mycobacteria-stimulated macrophages in vitro and in vivo as a model [16], in which comparably high and enhanced Wnt6 expression was observed by varieties of densitometry analysis, and an addition of exogenous Wnt6 led to moderately up-regulated Arginase-1 expression with a substantial increase in mannose receptor 1 (MRC-1, another well-annotated marker of alternative macrophage activation) production, which was accompanied by a decreased TNF*α* abundance. Of importance, a recent study focusing on COVID-19 patients has shown that the accumulation of ACE2 (angiotensin-converting enzyme 2), a primary cell receptor, is associated with macrophage activation and both IL-6 and TNF*α* overexpression, which is inversely correlated with the Wnt/*β*-catenin pathway [17].

As expected, Yang et al. [3] documented that Wnt3a inhibition using two structurally and functionally different chemical inhibitors, IWP-2 and PNU-74654, significantly enhances secretion of *Escherichia coli* LPS-induced IL-6, IL-12p40, and TNF*α* in human monocytes and THP-1 cell lines, which is also corroborated with a siRNA to knock down Wnt3a expression. Additionally, the addition of DKK1, a classical Wnt signaling inhibitor, showed an ability to reverse the Wnt3a-repressed TNF*α* and IL-6 expression in BCG-infected cell lines [13]. In consistence, silencing DKK1 markedly attenuated the inflammatory response to heat-inactivated *Rickettsia conorii* in HUVECs (human umbilical vein endothelial cells) with down-regulatory effects on IL-6, IL-8, and GRO*α*/CXCL1 at both transcriptional and translational level [18]. Likewise, blockade of the Wnt pathway using XAV939 reduced Wnt-target gene expression while promoting IL-1*β* transcription in a mouse model of endotoxemia [19].

In parallel, a recent series of studies by Liu et al. [20, 21] pointed out that infection with wild *Salmonella* SL1344 in vitro and in vivo was observed to cause an up-regulation of Wnt2 and Wnt11 expression at both transcriptional and translational levels. In this regard, AvrA, a well-known effector protein that is responsible for inhibiting Salmonella-induced intestinal inflammation by stabilizing *β*-catenin [22, 23], is shown to stabilize and enhance both Wnt2 and Wnt11 in a time-dependent manner via serving as a deubiquitinase [24]. A qRT-PCR assay revealed that after 3 weeks of infection, mice infected with the AvrA^−^ mutant had been induced with several essential pro-inflammatory cytokines, such as ICAM, IL-1*β*, TNF*α*, and MIP-2, as demonstrated by an in vivo study [25]. Of surprise, such induction was continuously detected up to 27 weeks of infection, with the AvrA^−^ infected group exhibiting notably higher concentrations of IL-1*β* and MIP-2 compared with the control [25], hence implying a potential part in controlling the prolonged interplay between the host and bacteria. Likewise, AvrA^−^ deficient strain showed a higher expression of IL-6 at both mRNA and protein levels, whereas complementation with AvrA in human embryonic kidney 293 cells colonized with AvrA^−^/AvrA^+^ diminished IL-6 production to the control levels similar to parental strains [24]. As expected, IL-8, a well-accepted inflammatory readout for epithelial–bacterial interaction, was significantly downregulated in the overexpressed Wnt2/Wnt11 system following *Salmonella* colonization [20, 21]. In comparison to the uninfected human colonic epithelial HCT116 cell line, Wnt1- knockdown cells displayed a substantial up-regulation of IL-6, IL-8, and granulocyte-macrophage colony-stimulating factor (GM-CSF) transcripts upon colonization by Salmonella [26]. Consistently, an ELISA assay showed an augmented IL-6 secretion in culture supernatant, although no evident changes were detected in both IL-8 and GM-CSF production [26]. Intriguingly, in contrast to the expectation that Wnt1 overexpression would induce less inflammation followed by *Salmonella* challenge, overexpression of Wnt1 has been indicated to positively affect *Salmonella*-induced inflammation [26]. These discrepant findings might be due to the complicated coordination effects of different Wnt members in response to bacterial infection. One possible explanation was that Wnt2 and/or Wnt11 expression were synergistically upregulated with the knockdown of Wnt1, while the expression of Wnt2 and/or Wnt11 was downregulated with the overexpression of Wnt1. Taken together, these findings suggested that the reduction of Wnt1 associated with Wnt2 and Wnt11 may serve as a characteristic strategy for host defense via negatively regulating *Salmonella*-induced intestinal inflammation.

In separate settings, administration of a GSK3*β* inhibitor SB216763 (mimic the activation of Wnt/*β*-catenin signaling) potently suppressed the TNF*α* expression in murine BMDMs in response to *M. avium* stimulation [12]. In murine peritoneal macrophages infected with an intracellular pathogen *F. tularensis* [27] and in human monocytes receiving lipopolysaccharide (LPS) [28], a marked rise in the production of anti-inflammatory cytokine IL-10 and a considerable decrease in pro-inflammatory cytokines TNF*α*, IL-6, and IL-12p40 were observed, when GSK3*β* was inhibited using an alternatively potent and selective inhibitor LiCl or 2Z, 3E-6-bromoindirubin-3-oxime (BIO). In consistence, treatment with LiCl in Avian infectious bronchitis virus(IBV)-induced BHK cell or IBV-infected chicken embryos significantly down-regulated the expression of inflammatory cytokines including TNF*α* and IL-1*β* in a dose-dependent manner [29]. Similarly, in Marek's disease virus (MDV)-infected chicken embryonic fibroblast (CEF) cells, there was increased expression of IFN-*β*, IFN regulatory factor 7 (IRF7), and OASL, as expected. However, these gene expressions were reduced significantly in LiCl-treated cells [30]. Investigation of LiCl's inhibitory effects on MARC-145 and PAM-CD163 cells infected with porcine reproductive and respiratory syndrome virus (PRRSV) revealed that, after treatment with LiCl, viral replication was decreased and mRNA levels of pro-inflammatory IL-8, IL-6, IL-1*β*, and TNF*α* were decreased, implying that LiCl moderate PRRSV-induced inflammatory responses via reducing the expression of pro-inflammatory cytokines associated with viral replication [31]. In consistence, cells transfected with GSK3*β* siRNA exhibited a significantly enhanced Wnt activity, along with diminished level of TNF*α* production, while the abundance of supernatant IL-10 was largely augmented [27]. Similar results were obtained by Huang et al. [32] in LPS-stimulated microglia, in which silencing GSK3*β* is responsible for up-regulating the production of IL-10, indicative of an anti-inflammatory microenvironment. Consistent with the above studies, treatment of either BIO or LiCl largely blocked the production of heat-inactivated *S. aureus*-induced TNF*α* in a time-dependent manner, partially attributed to the inactivation of NF-*κ*B signaling, whereas an augmented early expression of the immunosuppressive IL-10 was detected in BV-2 mouse microglia [33]. This finding was supported by a subsequent experimental system [34], in which blockage of GSK3*β* activity by different selective pharmacological inhibitors or its gene silencing by small interfering RNA attenuated LPS-induced TNF*α* production in cultured microglia. Additionally, GSK3*β* inhibition negatively regulated *Salmonella typhimurium*-triggered inflammatory responses in both HT29-CI.19A and T84 colonic epithelial cell lines via suppressing the secretion of pro-inflammatory cytokine IL-8, which was confirmed by an ELISA assay [35]. Besides, an in vivo study demonstrated that administering a GSK3*β* inhibitor to mice effectively reduced the serum IL-6 level by 67% compared to the untreated control following LPS treatment [36]. In contrast, constitutively active S9A-GSK3*β*, where a serine subject to inhibitory phosphorylation was mutated to alanine to prohibit the inhibitory phosphorylation, successfully resulted in an augmented IL-6 production in primary enriched astrocytes [36]. Altogether, these data supported that inhibition of GSK3*β* provides a pivotal control point in the modulation of pathogenic bacteria-triggered inflammatory cascade by differentially affecting the induction of pro- and anti-inflammatory cytokines and chemokines.

*β*-catenin is involved in Wnt signaling pathway and plays an indispensable role in the transmission of signals from the cell cytoplasm to the nucleus and is a critical intracellular transducer responding to various physiological and pathological processes. Nevertheless, whether *β*-catenin acts as a suppressor or activator in host inflammatory responses remains controversial. Current knowledge suggests that the infection-induced modulation of *β*-catenin was probably closely related to the pathogen specificity. In this regard, an antagonistic regulation between *β*-catenin and inflammation during bacterial infection will be described. *β*-catenin overexpression in murine macrophage-like RAW264.7 cells and bone marrow-derived neutrophils that were exposed to *P. aeruginosa* (PA) resulted in a decreased abundance of numerous pro-inflammatory cytokines at the transcriptional level including TNF*α*, IL-6, and MIP-2, despite there were no statistically significant differences in immunosuppressive IL-10 transcripts were detected between two different settings [37]. Chen et al. [37] demonstrated that *β*-catenin has a comparable anti-inflammatory role; they observed a significant suppression of PA infection in corneas treated with *β*-cat-lentivirus. IL-1*β*, TNF*α*, and MIP-2 production at the transcriptional level and a considerable decrease of both IL-1*β* and IL-6 production at the translational level in contrast to Ctl-lentivirus treated group, indicating that *β*-catenin serves as an inflammatory inhibitor in PA keratitis. Besides, Lectin LecB, a virulence factor of *P. aeruginosa*, was mainly located at the outer bacterial membrane, responsible for biofilm formation [38]. LecB stimulation down-regulated the nuclear translocation of *β*-catenin in vitro via inducing its proteasomal degradation, as seen in immunofluorescence experiments as well as in western blot analysis, along with a substantial and continuous up-regulation of a pro-inflammatory cytokine TNF*α*, whose transcript was elevated up to above 20-fold after overnight incubation [39]. This LecB-induced repression of *β*-catenin and production of TNF*α* would be expected to be via a GSK-3*β*-dependent way. Notably, LecB coincubation with Wnt3a was sufficient to prevent the accumulation and nuclear translocation of *β*-catenin caused by Wnt3a [39], indicating a crosstalk between Wnt3a and LecB in controlling bacteria-induced inflammation. A study corroborated this idea, showing that the overexpression of dominant-active *β*-catenin in human alveolar type II epithelial cells (A549) had anti-inflammatory properties that were exposed to BCG or LPS resulted in a reduced TLR signaling with a considerable decrease in TRAF6 and NF-*κ*B expression, followed by a battery of suppressed IL-1*α*, IL-2, IL-6, IL-8, and TNF*α* production [40]. Similarly, overexpression of *β*-catenin mutant may prevent Wnt3a inhibitor from enhancing inflammatory cytokines stimulated by LPS in human monocytes [3]. Using a gene mutation assay, it was also discovered that *Salmonella*-induced IL-8 production was eliminated in human intestinal epithelial cell lines HCT116 that overexpressed constitutively active *β*-catenin [41].

For another, the suppression of pro-inflammatory cytokines' mRNA level expression due to LiCl in PRRSV infection was reversed when *β*-catenin's expression was silenced [31]. Using prevalidated siRNA for *β*-catenin, researchers found that treatment of cells with LPS alone resulted in significant increases of IL-12P40, IL-6, and TNF*α* compared to the control group, which mimics the effect of Wnt3a inhibitor [3]. Consistent with the results described above, significant augmented NF-*κ*B activity was observed in *β*-catenin siRNA-transfected LPS-stimulated RAW264.7 cells, which was accompanied by a robust elevated mRNA level of IL-6 [42]. Also, treatment of *β*-catenin inhibitor iCRT14 up-regulated the expression of interferon-related genes, such as IFN*β*, IRF7, and OASL, as well as IL-8 in CEF cells infected with MDV, indicating an anti-inflammatory effect of *β*-catenin [30]. The anti-inflammatory role of *β*-catenin was parallelly clarified in *Haemophilus parasuis*-infected acute systemic inflammation. He et al. [43] showed treatment of PK-15 and NPTr cells with the selective *β*-catenin inhibitor IWR-1-endo dramatically enhanced *H. parasuis*-induced IL-8, CCL4, and CCL5 expression at the transcriptional level. Another unpublished article has shown the expression of pro-inflammatory factors including TNF*α*, IL-1, and IL-8 in FH535 (a specific *β*-catenin inhibitor) treated Qi river crucian carp (*Carassius auratus*) were significantly elevated upon *Aeromonas hydrophila* infection, indicating *β*-catenin may play a key role in anti-inflammatory response. Furthermore, an analysis of DCs functions in a mouse model of warm liver ischemia and reperfusion injury (IRI) provided backing for these results; *β*-catenin signaling being depleted, hepatic DC maturation was augmented, and local inflammation followed suit, as well as mRNA levels coding for IL-6, IL-12p40, TNF*α*, and CXCL-10 [44]. The anti-inflammatory properties of *β*-catenin were highlighted by such data, and it was found that DCs are regulated to a tolerogenic state by *β*-catenin, thus restraining the pro-inflammatory reaction caused by bacteria.

The evidence, taken together, implies a repressive impact of the Wnt/*β*-catenin pathway on pro-inflammatory responses in bacteria, varying from cell to cell (Table 1); however, the underlying molecular mechanisms by which Wnt signaling components control inflammation remain to be further explored.

## 4. Negative Regulation of Inflammation by Different Components of Wnt Signaling

It has been shown that canonical Wnt/*β*-catenin signaling was required for the progression of anti-inflammatory responses; thus, current studies focus on whether Wnt signaling suppresses the production of pro-inflammatory mediators directly or in an indirect manner via regulating other inflammation-associated signaling pathways, and which components serve as determinants in response to inflammation and infection. With this in mind, the underlying molecular mechanisms of GSK3*β* and *β*-catenin involved in Wnt-modulated anti-inflammatory effects are respectively discussed.

### 4.1. GSK3*β* Inhibition Suppresses Inflammatory Responses

#### 4.1.1. Via Stabilization of I*κ*B*α*

The family of inducible transcription factors known as nuclear factor-*κ*B (NF-*κ*B) is a critical downstream mediator of immediate early gene expression, linking extracellular signals to gene-activating events often associated with pro-inflammatory reactions. Comprising a family of inducible transcription factors, NF-*κ*B is typically composed of RelA/p65, c-Rel, RelB, p50 (NF-*κ*B1), and p52 (NF-*κ*B2). This inactive form is kept sequestered within the cytoplasm by its physical association to an inhibitory kappa B (I*κ*B) member [45]. Posttranslational mechanisms related to the dissociation of I*κ*B complex are responsible for the phosphorylation and translocation of NF-*κ*B, a characteristic feature [46, 47]. Several lines of evidence have suggested a close-knit linkage between GSK3*β* and NF-*κ*B signaling in regulating bacteria-triggered inflammation. Herein, inhibition of GSK3*β* can alter NF-*κ*B activity, and this cross-regulation occurs via *β*-catenin and I*κ*B*α* [35, 48]. A recent study in cloned osteoblast-like cells (MC3T3-E1) has shown that inactivation of GSK3*β* with SB216763 or siRNA-transfected cells triggered a substantial decrease in phosphorylation of I*κ*B and subsequent nuclear RelA/p65 protein expression upon LPS stimulation, with was concurrently confirmed by western blot and NF-*κ*B DNA-binding assays [48]. Moreover, pretreatment of SB216763 in LPS-stimulated MC3T3-E1 cells activated Wnt/*β*-catenin signaling by an increasing translocation of *β*-catenin to the nucleus, with was performed by an immunofluorescence experiment, in which nuclear staining of RelA/p65 was barely invisible [48]. In parallel, HCT116 cells transfected with siRNA for GSK3*β* showed stabilization of both I*κ*B*α* and *β*-catenin in response to WT *Salmonella* colonization, which was accompanied by an attenuated IL-8 transcript [35], indicative of an I*κ*B*α*-related suppression effect by GSK3*β* inhibitor treatment or GSK3*β* knockdown. Comparably, less WT *Salmonella*-induced IL-8 secretion was detected in the supernatant of polarized human colonic epithelial cell lines HT29Cl.19A and T84 following LiCl treatment, which might be partially attributed to the increased I*κ*B*α* stabilization. It is conceivable that I*κ*B*α* is degraded in a similar manner with *β*-catenin as a result of shared GSK3*β* activity, followed by increased NF-*κ*B translocation and target gene expression. However, it has been recently documented that LPS stimulation caused the phosphorylation of both GSK3*β* and NF-*κ*B, as well as a change in total I*κ*B expression. In contrast to the augmentation of total I*κ*B, the increased phosphorylation of GSK3*β* was slightly postponed [3], implying that other downstream molecules, not I*κ*B, were employed by GSK3*β* to impede NF-*κ*B's activity. Similarly, the presence of a GSK3*β* inhibitor resulted in an expectable downregulation of NF-*κ*B-regulated gene transcription without affecting the degradation or re-synthesis of I*κ*B*α* [49, 50]. The potential for these contentious discoveries to be, at least in part, the consequence of varying GSK3*β* compartmentalization on signaling pathways [51], as well as the distinctions in stimuli types, cell environment, and experimental-dependent methods, is uncertain. Consequently, additional I*κ*B*α*-independent manners may be involved in GSK3*β* inhibition-associated anti-inflammatory response.

#### 4.1.2. Via Bind of CBP to CREB

The regulation of NF-*κ*B is characterized at multiple levels: synthesis, nuclear localization, differential dimerization, posttranslational modification, DNA binding, and interaction with a specific combination of coactivators. A histone acetyltransferase p300 or its homolog, CREB-binding protein (CBP), is considered as a key coactivator tightly associated with NF-*κ*B activation via a physical association with RelA/p65 subunit. Nevertheless, as a matter of fact RelA/p65 interacts with CBP/p300 at the same region as phosphorylated CREB (cAMP response element-binding protein) [52]. Consequently, an increased association of CREB and CBP/p300 results in a considerable inhibition of RelA/p65 activity via competition for limiting nuclear amounts of CBP/p300. Martin et al. [28] conducted a groundbreaking investigation into the ways in which GSK3*β* deactivation had an adverse effect on the inflammatory reaction of LPS-stimulated monocytes. They demonstrated that when SB216763 was used as a selective pharmacological inhibitor to reduce GSK3*β* activity, it significantly augmented CREB's DNA binding properties, yet remarkably repressed NF-*κ*B-mediated pro-inflammatory responses by varying the interaction between nuclear RelA and CREB with coactivator CBP/p300, therefore clarifying an ability of GSK3*β* to mediate immune responses is, at least in part, dependent on regulation of CREB activity. A knockdown assay revealed siRNA specific to CREB, and when stimulated with LPS, IL-12p40 production was observed to be 20% higher than that of un-transfected cells [28], further confirming the mechanisms.

#### 4.1.3. Via Up-Regulation of IL-10 Expression

IL-10 is a well-documented signature that contributes to host immune inhibitory bioactivities. Recent studies have suggested that GSK3*β* could be a potential negative regulator of IL-10 production in various cell lines, such as human monocytes [28], murine macrophages [53], and human B cell lines [54], in contrast to the majority of signaling molecules which regulate IL-10 production being positive regulators to date. Nevertheless, only scanty information is available on how pathogenic bacteria-triggered IL-10 hyperinduction is mediated by a GSK3*β* inhibition. The essential role of phosphorylated CREB in the production of IL-10, and its subsequent inhibition of amplification loops in the inflammatory cascade, has been corroborated by multiple lines of evidence [53]. Herein, to determine whether GSK3*β* inhibition-mediated IL-10 introduction is dependent on CREB activation, supernatants of LPS-stimulated monocytes pretreated with siRNA targeting CREB were analyzed by immunoblot and ELISA. A marked decrease of more than 30% in IL-10 release was revealed in comparison to the untransfected group [28]. However, as anticipated, no discernible increase in immunosuppressive IL-10 production was observed in the presence of SB216763 [28], indicating that CREB activation may be a downstream target of GSK3*β* inhibition responsible for mediating an anti-inflammatory effect. Of particular note, the depletion of CREB failed to completely block the production of IL-10, suggesting an involvement of additional mechanisms in place, which can induce quite IL-10 transcription in the absence of CREB.

Recently, a new elucidation of the augment of IL-10 activation was presented, based on the evidence that GSK3*β* inhibition dephosphorylated and unstabilized p105 [55]. In contrast to the wild-type group, cells without GSK3*β* processed the p105 precursor to the p50 subunit at a faster and more accelerated rate, yet this rate could be brought back to a baseline when GSK3*β* was reintroduced by transfection. Emerging evidence has supported the critical role of the p50 subunit in positively driving IL-10 transcription [56, 57]. On account of the absence of a transactivation domain, p50 forms a homo-/hetero-dimer to be transcriptionally active, in which p50/p50 or p50/p105 are commonly supposed to be transcriptional repressors, and are the only NF-*κ*B family member contributing to drive IL-10 transcription. Mechanistically, p50 binds to the specific site in the proximal IL-10 promoter and triggers its transcriptional activity. This opinion is supported by a series of related findings in bacteria-associated experimental systems. Cao et al. [56] showed that BMDMs from mice lacking p50 pretreated with LPS decreased amounts of IL-10 at both transcriptional and translational levels relative to wild-type mice, along with a moderate and robust up-regulation of IL-12 and TNF, respectively; however, a transfection of reconstitute p50 expression construct were sufficient to induce a recovery of IL-10 production in response to LPS stimulation. In line with this notion, the lack of a p50/p105 subunit in hemopoietic cells in vivo impedes IL-10's capacity to suppress *Helicobacter hepaticus* (Hh)-induced innate inflammation, thus making NF-*κ*B-deficient mice more vulnerable to Hh-associated colitis [57]. Additionally, suppression of IL-10 target genes such as IL-12p40 is markedly compromised in macrophages lacking p50/p105 subunits of NF-*κ*B following Hh or LPS treatments [57]. Altogether, these findings strongly suggested a noteworthy and alluring part of GSK3*β* in thwarting bacterial evasion of host immunity through IL-10 expression. Nevertheless, apart from CREB and p50 regulation, the consequences of inhibiting GSK3*β* on the other transcription factors that positively regulated IL-10 production should be further investigated.

#### 4.1.4. Via Up-Regulation of IL-1Ra Expression

The IL-1 receptor antagonist, IL-1Ra, is believed to be a major contributor in demonstrating powerful anti-inflammatory effects and providing protection against inflammatory-associated diseases [58, 59], due to its endogenous inhibition of pro-inflammatory cytokine IL-1*β*. siRNA or LiCl pretreatment data suggested that GSK3*β* inactivation remarkably up-regulated IL-1Ra expression in LPS-stimulated human monocytes while concurrently suppressing IL-1*β* secretion [60]. The utilization of a specific inhibitor U0126 could almost completely eradicate the phosphorylation of extracellular-signal-regulated kinase 1/2 (ERK1/2), thus affirming the significance of ERK1/2 signaling in both IL-1Ra and IL-1*β* production's positive and negative modulation, respectively [60]. However, another seminal study by Molnarfi et al. [61] has revealed that a loss of ERK1/2 activity resulted in the synergistic reduction of both IL-1Ra and IL-1*β*, thus indicating an involvement of additional anti-inflammatory mechanisms apart from ERK1/2 signaling. Accordingly, it is conceivable that the ability of GSK3*β* inhibition to increase ERK1/2 phosphorylation while collaboratively attenuating NF-*κ*B activation is likely responsible for its ability to differentially regulate the expression of IL-1Ra and IL-1*β*, eventually limiting overwhelming inflammation.

**Figure 2:**
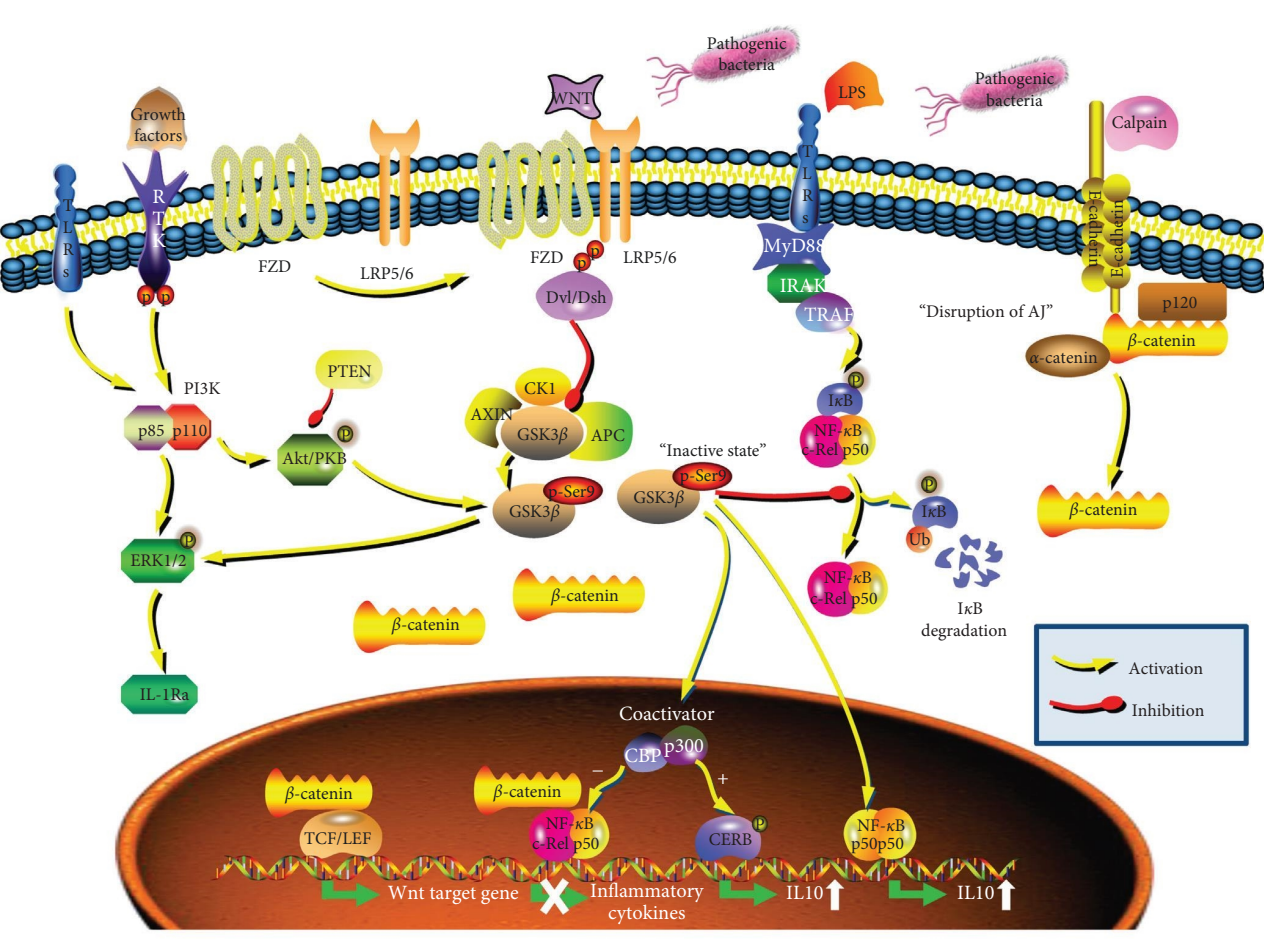
Scheme shows possible mechanisms of canonical Wnt/*β*-catenin pathway in the regulation of pathogenic bacteria-induced inflammatory responses. The figure represents an essential role of Wnt/*β*-catenin signaling serving as an inflammatory suppressor via several possible mechanisms: limitation of the expression of pro-inflammatory cytokines by sequestering NF-*κ*B c-Rel/p50 signaling [3, 35, 41, 48, 62–64], promotion of the expression of CREB- and p50/p50-dependent immunosuppressive IL-10 [55–57], and up-regulation of the expression of IL-1Ra via an ERK1/2-dependent manner [58–61].

Altogether, these findings eloquently elucidated the molecular mechanisms whereby GSK3*β* inhibition prevents pro-inflammatory cytokines expression but augments the production of anti-inflammatory mediators, thus providing supportive evidence of the potential of GSK3*β* inhibition in suppressing the host immune responses in pathogenic bacteria-infected inflammatory diseases (Figure 2). As a result, GSK3 maybe a novel remedy for pathogenic bacteria infections or diseases that necessitate a harmony between pro- and anti-inflammatory cytokine productions.

### 4.2. *β*-Catenin Activation Suppresses Inflammatory Responses

#### 4.2.1. Via as a Negative Regulator of NF-*κ*B Activity

Current knowledge has suggested an essential role of *β*-catenin serving as an inflammatory mediator via a major possible mechanism: suppressing the expression of pro-inflammatory cytokines by sequestering NF-*κ*B signaling. Biochemical and genetic approaches have suggested that the cross-regulation of *β*-catenin and NF-*κ*B signals is a pivotal factor in controlling pathogenic bacteria-induced inflammation [65]. Simultaneously with the augmentation of *β*-catenin accumulation, Yang et al. [3] showed that NF-*κ*B phosphorylation diminishes; moreover, when *β*-catenin was overexpressed, Wnt3a inhibition had no effect on NF-*κ*B's DNA binding capacity, implying that *β*-catenin could be a hindrance to NF-*κ*B activity in LPS stimulated monocytes. Mounting evidence has indicated that the *Salmonella* strain PhoP^c^, which is not virulent, may reduce NF-*κ*B-related pro-inflammatory responses by hindering *β*-catenin breakdown, thus permitting its accumulation and translocation into the nucleus. The induction of NF-*κ*B activity through *β*-catenin degradation upon *P. aeruginosa* infection also explains why LecB-mediated effects on NF-*κ*B are delayed compared to the instantaneous activation of NF-*κ*B by other molecules such as TNF*α* [39]. A *β*-catenin system, constitutively active, could be used to explain why *Salmonella*-induced I*κ*B*α* protein was stabilized, leading to NF-*κ*B's inability to separate from I*κ*B*α* and the cytoplasmic retention of NF-*κ*B. In derivative HCT116 cell lines, a negative regulation of NF-*κ*B signaling by *β*-catenin was observed; when the latter was not present in its original form, I*κ*B*α* degradation was significantly increased. However, constitutively active *β*-catenin was found to reduce I*κ*B*α* degradation by twofold [41]. In agreement with the prior notion that *β*-catenin is phosphorylated in response to pathogenic bacteria, and I*κ*B*α*, the negative regulator of NF-*κ*B, is degraded similarly to p-*β*-catenin, NF-*κ*B infiltrates the nucleus and amplifies pro-inflammatory cytokine secretion, thus inducing inflammation [35]. The coimmunoprecipitation assay data revealed that *β*-catenin had no physical interaction with I*κ*B*α* [41], indicating that I*κ*B*α* may be stabilized by an alternative mechanism that is not dependent on direct *β*-catenin binding. Intriguingly, Wnt3a stimulation of *β*-catenin's physical interaction with p50 and RelA/p65 was observed in a variety of cell types such as fibroblasts [62], epithelial cells [35, 41], osteoblasts [48], hepatocytes [63, 64], and so on along with a reduction in NF-*κ*B reporter activity. By performing an immunoprecipitation assay in MC373-E1 cells after exposure to LPS for 24 hr, a remarkably augmented *β*-catenin and RelA/p65 complex were detected in the presence of SB216763; however, silencing *β*-catenin by specific siRNA decreased the amount of *β*-catenin pulled down by RelA/p65 and restored the diminish of nuclear RelA/p65 translocation and its DNA-binding activity [48]. Following *Salmonella* colonization, a direct characterization of the protein–protein interaction between *β*-catenin and p50 was conducted in an in vitro pulldown assay using only recombinant proteins of both signatures [41]. These findings provided supportive shreds of evidence that the convergence of *β*-catenin and NF-*κ*B pathways is due to the direct interaction between NF-*κ*B RelA/p65 or p50 subunits and *β*-catenin.

Additionally, repression of NF-*κ*B activity by *β*-catenin may be attributed to the decreased CBP-mediated acetylated effects for RelA/p65. In this perspective, overexpression of *β*-catenin or Wnt3a costimulation was found to reduce acetylation of RelA/p65 protein levels, and that of diminished acetylation is nuclear CBP-dependent, since knockdown of CBP in primary human lung fibroblasts MRC-5 reversed the down-regulated pro-inflammatory subset of NF-*κ*B target genes [62]. The regulatory effect of *β*-catenin on RelA/p65 posttranslational modifications, in contrast to blocking the entire nuclear NF-*κ*B translocation that encompasses all downstream target genes, could offer a more elaborate and selective control (Figure 2).

#### 4.2.2. Via a Negative TLR4 Regulatory Feedback

The activation of *β*-catenin in murine BMDCs stimulated by LPS, in line with its detrimental regulatory role in NF-*κ*B activities, was found to suppress TLR4-driven inflammation and, eventually, reduce the production of pro-inflammatory cytokines, such as IL-12 [44]. The deletion of inhibited phosphatase and tensin homologs on chromosome 10 (PTEN) activation was observed in this context, yet provoked Akt phosphorylation was detected afterward, suggesting a noteworthy part the PTEN/Akt pathway plays in controlling *β*-catenin activity. A mouse model of warm liver IRI revealed analogous outcomes: transfection of siRNA caused *β*-catenin disruption, thus inhibiting transcription of its target genes, stimulating PTEN activity, and diminishing the PI3K/Akt pathway, which in turn programed dendritic cells (DCs) to an active state. Remarkably, upon *β*-catenin knockdown, I*κ*B*α* phosphorylation, and ubiquitination were both increased, as well as a pro-inflammatory gene program driven by TLR4, implying that *β*-catenin may have an effect on TLR4 signaling through a negative feedback regulatory process. It has been proposed that *β*-catenin acts as a pivotal mediator to bridge the crosstalk between pro- and anti-inflammatory pathways and served as a pivotal determinant in the maintenance of homeostasis in infected organisms, where active *β*-catenin signaling represses a pro-inflammatory response and protects the host against overwhelming inflammation. Such findings provide a rationale for fresh therapeutic approaches to control local inflammation upon bacterial infection.

#### 4.2.3. Via Up-Regulated Expression of Immunosuppressive Factor

Alternatively, emerging evidence has demonstrated that *β*-catenin acts as an inflammatory inhibitor by inducing secretion of potent immunosuppressive substances, such as IL-10, prostaglandin E2 (PEG_2_), and Cyclooxygenase-2 (COX-2), therefore limiting the host inflammatory response. It has been determined that COX-2 is a downstream gene of the Wnt/*β*-catenin signaling pathway [66]. Mounting evidence has demonstrated that expression of COX-2 is highly induced in response to inflammation, consequently repressing the pro-inflammatory cytokines, which means an anti-inflammatory loop triggered by COX-2 is involved during bacterial infections. He et al. [43] have shown that the expression of COX-2 was augmented by *H. parasuis*-induced Wnt/*β*-catenin activation, and overexpression of COX-2 diminished NF-*κ*B's activity via significantly attenuating p65 phosphorylation and subsequently the secretion of pro-inflammatory cytokines. Expectedly, the knockdown of COX-2 by siRNAs significantly increased the phosphorylated p65, suggesting that NF-*κ*B activation was negatively modulated by COX-2 expression triggered by Wnt/*β*-catenin signaling during *H. parasuis* infection. The inhibition of *β*-catenin activity pharmacologically almost completely eradicated the expression of COX-2, triggered by BCG and PEG_2_ secretion, thus further affirming that the interplay between the Wnt/*β*-catenin and NF-*κ*B signaling pathways mediated by COX-2 is a crucial factor in controlling host inflammation during bacterial infection.

In addition, *β*-catenin's role as an inflammatory inhibitor is demonstrated by its direct stimulation of the secretion of anti-inflammatory cytokine IL-10 by CREB. Phosphorylated CREB is capable of binding to IL-10 promotors with cAMP response elements, which then leads to a heightened expression of IL-10 [67]. In this perspective, blockage of *β*-catenin/CREB-binding protein (CBP) interactions by ICG-001 effectively decreased the serum IL-10 level [52], supporting such a conclusion.

Altogether, the possible mechanisms of *β*-catenin that could impede uncontrolled inflammation and be advantageous for anti-infection immunity were outlined; however, a more precise mechanism of its association with microbial infection has not been well elucidated. Consequently, further research into the precise characterization of *β*-catenin will aid us in gaining a more comprehensive comprehension of the Wnt signaling-modulated anti-inflammatory reactions.

## 5. Regulation by Other Signalings Links Anti-Inflammation

The complexity of the regulatory network, a result of the diversity and homeostasis inherent in biological systems, is amplified by the interplay between signaling pathways, thus extending the functions of individual pathways. Accumulating evidence has suggested that Wnt/*β*-catenin signaling exerts its anti-inflammatory property via a wide crosstalk with other signaling pathways. Very little, however, is known about whether the inflammatory milieu contributes to the promotion of Wnt/*β*-catenin signaling. Here, the intricacy of Wnt/*β*-catenin, regulated by other signals to connect it to the biological significance of pathogen-triggered inflammation, will be elucidated.

### 5.1. Regulation by PI3K/Akt Pathway

The roles of the PI3K/Akt pathway in mediating inflammation-associated gene expression are controversial. Up-to-date publications have described that PI3K is a component of an intracellular control system governing the initial stages of the innate immune reaction to various microbial pathogens. Since PI3K/Akt and Wnt/*β*-catenin signaling share critical effectors, such as GSK3*β*, receptor frizzled (FZD), and adaptor disheveled (Dvl) that are engaged in both pathways [68], it is speculated that there may be an interaction between the two pathways to jointly complete the regulation of inflammation. In most situations, PI3K/Akt signaling has been shown to act negatively on inflammatory gene expression by revitalizing anti-inflammatory signaling pathways or suppressing pro-inflammatory signaling pathways. In this regard, blockage of PI3K activity by a pharmacological inhibitor LY294002 in human PBMCs had a detrimental effect on *M. bovis* BCG-induced Akt (Ser473) and GSK3*β* (Ser9) phosphorylation, along with a substantial down-regulation of IL-10 production [69]. In consistency, inhibition of PI3K or Akt using LY294002 or Akt inhibitor, respectively, abolished *E. coli* LPS's capacity to deactivate GSK3*β* by phosphorylating Ser9, thus rendering the kinase in its active state, resulting in a heightened production of pro-inflammatory cytokine IL-12p40 and a notably decreased expression of anti-inflammatory cytokine IL-10 in human monocytes [28]. The PI3K inhibitor wortmannin and cells transfected with siRNA targeting Akt yielded similar outcomes, affirming that PI3K/Akt, acting as a negative regulator of GSK3*β*, had a positive effect on Wnt activity and that this gave rise to an anti-inflammatory surrounding milieu upon bacterial infection. The phosphorylation of the N-terminal serine of GSK-3*β*, due to Akt activation, can partially account for these results; this inhibits the activity of GSK-3*β* and leads to the separation of *β*-catenin from the complex [70].

Besides, the toxin VacA is generally believed to be responsible for the pathogenesis and severity of *H. pylori*-associated disease. The inhibitory effects of LY294002 and PI3K silencing blocked VacA-induced GSK3*β* phosphorylation in human gastric adenocarcinoma AZ-521 cells, subsequently resulting in a decreased relative abundance of nuclear *β*-catenin, thus suggesting activation of Wnt signaling might be at least partially dependent on PI3K/Akt pathway. In a separate setting, the inhibition of PI3K activity in a distinct environment had a beneficial effect on LPS-triggered inflammatory responses in human PBMCs, via down-regulation of Wnt activity and subsequent IL-1Ra production [60], accordingly hastening the release of pro-inflammatory cytokines, hence providing a rationale for regulating the nature and severity of inflammation. In agreement, administration of Akti-1/2 (a specific Akt inhibitor) in both MDCK and AGS cells potently restricted GSK3*β* phosphorylation, therefore relating to regulation of *β*-catenin signaling in *H. pylori*-induced cells, and this response was further confirmed by the performance of a siRNA transfection experiment, in which anti-Akt siRNA prevented inhibitory GSK3*β* phosphorylation, making for the restoration of *β*-catenin phosphorylation. The crucial role of Akt/GSK3*β* and Ser/Thr phosphorylation of *β*-catenin in the regulation of *β*-catenin nuclear activity during *H. pylori* infection [71] is evidenced by Akti-1/2′s inhibition of LEF/TCF transactivation in HP-infected cells, in addition to restoring *β*-catenin phosphorylation. Comparably, Akt has been showed to phosphorylate *β*-catenin directly at a distinct site independent of GSK3*β*, increasing its nuclear translocation and transcriptional activity [72]. Similarly, Akt increases the phosphorylation of *β*-catenin at Ser552 leading to the stabilization and nuclear translocation of *β*-catenin, thus implying that *β*-catenin is activated downstream of PI3K/Akt. A potential mechanism is that Akt negatively controls FOXO1a activity through phosphorylation, causing its export from the nucleus, sequestration in the cytoplasm, and degradation by the proteasomal machinery, thus increasing *β*-catenin transcriptional activity [73]. Specifically, FOXO1a may usually antagonize *β*-catenin via binding to it, preventing it from regulating certain transcriptional targets. The PI3K/Akt pathway's activation, and the resultant export of FOXO1a from the nucleus, could potentially inhibit *β*-catenin, thus allowing it to interact with NR5A1 on the Cyp19a1 promoter [74]. Alternatively, phosphorylation of EZH2 (zeste homolog) by AKT induced EZH2 to interact with and methylate *β*-catenin at lysine 49, which yielded *β*-catenin to interact with TCF1 and RNA polymeraseⅡ, thus fine-tuning *β*-catenin's transcriptional activity and resulting in the promotion of Wnt signaling [75], which indicated that Akt activation promotes *β*-catenin-mediated up-regulation of Wnt signaling in an EZH2-dependent manner. This evidence supports our view that the PI3K/Akt signaling pathway is a crucial cell survival route and can further augment the anti-inflammatory effects of pathogenic bacteria infection via the Wnt/*β*-catenin signaling pathway and its related downstream genes. Therefore, the interplay between the PI3K/AKT and Wnt/*β*-catenin pathways is essential, and therapeutic strategies targeting major proteins for the PI3K/AKT and Wnt/*β*-catenin pathways may influence anti-inflammatory responses.

Intriguingly, in contrast to the study in which the activation of the Wnt and PI3K/Akt signalings made collaboration to augment *β*-catenin activity in response to pathogen challenge, Brown et al. [76] described that inflammatory signals derived from the surrounding milieu did indeed induce *β*-catenin signaling in a PI3K/Akt-dependent manner, followed by the colonization of *Citrobacter rodentium*; however, no statistically significant differences in the induction of pro-inflammatory mediators were observed duringPI3K inhibition. In addition, inhibition of PI3K or Akt by LY294003 and Akti-1/2, respectively, was insufficient to influence *P. aeruginosa* LecB-induced *β*-catenin degradation, whereas such degradation was able to be accomplished to some extent in the presence of LiCl [39]. A possible explanation is that lecB induced proteasomal degradation of *β*-catenin via a GSK3*β*-mediated way, independent of PI3K/Akt signaling, facilitating the establishment, and stabilization of bacterial infection. Together, these findings argued that additional parallel pathways were involved in regulating Wnt-associated inflammation.

### 5.2. Regulation by TLRs/NF-*κ*B Pathway

As described previously, inhibition of GSK3*β*, as well as activation of *β*-catenin provide key control points in the inflammatory cascade via partially targeting NF-*κ*B. The bidirectional nature of the cross-regulation between Wnt/*β*-catenin and NF-*κ*B signalings in most biological processes has been revealed, with both pathways reciprocally regulating one another. It has been suggested that TLR signaling could modify the expression and action of the Wnt signals, thus sustaining the equilibrium of TLR-mediated inflammatory reactions. Yang et al. [3] described that TLR4 activation enhanced the expression of Wnt3a and Dvl3, both of which function as negative regulators of TLR4-mediated inflammation via suppression of NF-*κ*B activity. Mechanically, since phosphorylation of GSK3*β* and subsequent accumulation of *β*-catenin is mediated by LPS-induced PI3K activation [77], it is possible that accumulation of TLR4-mediated *β*-catenin resulted from both PI3K and Wnt3a signaling. The increase of Wnt3a and its downstream canonical signaling components occurred in the early stage of LPS stimulation [3], suggesting TLR4-enhanced Wnt3a-Dvl3 could play a critical role in anti-inflammatory mechanism to restrict the excess inflammation and subsequent collateral tissue damages it inflicted. Of note, no significant augmentation of Wnt3a or Dvl3 mRNA was observed upon the LPS challenge [3]. Consequently, upregulation of Wnt3a and Dvl3 expression upon LPS stimulation may be via suppression of their ubiquitination, especially considering a plethora of ubiquitination E3 ligase could be activated by LPS in innate cells.

In parallel, a recent study showed in vitro infection of murine BMDMs derived from TLR2-deficient mice with *M. avium* caused a powerful decrease in FZD1, which was demonstrated to be responsible for Wnt3a-triggered cellular responses [12]. In similar experiments, FZD1 mRNA in MyD88-deficient macrophages was reduced by approximately 85% in responses to *M. avium* infection. As anticipated, a significant decrease in FZD1 transcript level was observed in *M. avium*-stimulated BMDMs in the presence of a specific NF-*κ*B inhibitor (BAY11-7082), and similar results were observed during *M. tuberculosis* challenge as well [12]. These findings strongly indicated that induction of FZD1 may be partially dependent on TLRs, MyD88, and a functional NF-*κ*B, and that inflammation-induced up-regulation of FZD1 renders macrophages sensitive to Wnt3a signals, thus suppressing the intense inflammation.

For another, apart from the role of a downstream effector in Wnt signaling, *β*-catenin can serve as a structural component. It has been reported that *β*-catenin participates in the formation of adherens junctions (AJs) under physiological conditions, thereby blocking the invasion of pathogenic bacteria into host cells [78]. The activation of Wnt signaling is dependent upon the E-cadherin/*β*-catenin cell adhesion complex's alternating components, which are closely linked to the nuclear translocation of *β*-catenin [79, 80]. In this perspective, O'Connor et al. [81] found that the infection of *H. pylori*-induced a cleavage and redistribution of E-cadherin, resulting in the internalization of *β*-catenin in human gastric epithelial MKN45 cells, and subsequent activation of Wnt signaling. Such Wnt activation has been demonstrated to be TLR2-dependent, in which enhanced calpain activity and rearrangement of E-cadherin and *β*-catenin were observed in cells pretreated with a TLR2 agonist P3C [81]. Additionally, blockage of TLR2 in MKN45 cells by administration of a neutralizing antibody was sufficient to prevent calpain activation and down-regulate Wnt/*β*-catenin signaling, strengthening the notion that inflammation-associated TLRs contribute to the promotion of Wnt/*β*-catenin signaling during bacteria colonization.

Of significance, the experiments utilizing TLR2- or MyD88-deficient BMDMs revealed that FZD1 can be activated without TLRs/MyD88 signaling, with NF-*κ*B-dependent and -independent TNF*α* being involved. In this regard, expression of FZD1 in TNF^−/−^ macrophages challenged with *M. avium*, *M. tuberculosis*, and LPS were almost completely abrogated, suggesting that TNF is an essential inducer of Wnt3a-mediated signaling in macrophages due to its ability in microbially induced FZD1 expression [12]. In consistence, pretreatment of cells with a neutralizing antibody against TNF*α* remarkably suppressed the effect by the addition of a conditional medium to promote Wnt activity upon *H. pylori* or LPS colonization [82, 83]. Comparably, the blockage of either TNFR1 or TNFR2 receptor by neutralizing antibody exhibited an ability to alleviate expression of FZD1 and elevate phosphorylation of *β*-catenin, resulting in TNF*α*-induced Wnt/*β*-catenin suppression [12, 82]. In a different setting, however, TNF production in supernatants from Wnt3a-pretreated macrophages was dramatically reduced by barely half during persistent *M. tuberculosis* infection, and that decrease was observed in a Wnt3a dose-dependent manner [12]. The expression of TNF in *M. avium*-infected macrophages was found to be dose-dependently decreased with the addition of SB216763, a widely employed stimulator of Wnt signaling [12], which is in agreement with this discovery. The abovementioned results suggested that there might exist a particular pathway, where macrophage-derived TNF, under pathogen infection, induced macrophages to produce and secrete Wnt receptor, such as FZD1, via reacting with TNF receptor, and in turn, hyperactivated Wnt signaling inhibited TNF expression in a negative feedback mechanism.

### 5.3. Regulation by MAPKs Pathway

The expression of mitogen-activated protein kinase (MAPKs) is thought to be a factor in the control of multiple signal transduction pathways, such as cell proliferation, migration, differentiation, autophagy, apoptosis, and inflammation [84]. At least three distinct groups of Ser/Thr protein MAPKs, which are evolutionarily conserved, can be distinguished: extracellular signal-regulated kinase (ERKs), Jun N-terminal kinases (JNKs), and P38 MAPKs. Recent advances suggest a possible intersection cross-reacting network between the MAPKs and Wnt/*β*-catenin pathway.

Ding et al. [85] demonstrated that the ERKs pathway provides a mechanic link for HBV-induced *β*-catenin stabilization. Upon HBV infection, ERKs activate GSK3*β* through a docking motif (^291^FKFP) located at the C terminus, phosphorylating it at the ^43^Thr residue. This then triggers p90RSK, a downstream kinase of ERKs, to phosphorylate GSK3*β* at Ser9, resulting in the inactivation of GSK3*β* and the eventual buildup of *β*-catenin. In agreement with this notion, activation of JAK/STAT3 associated with IL-6 enhanced the phosphorylation of ERKs, resulting in the upregulation of *β*-catenin [86]. Besides, ERKs activate Wnt signaling by phosphorylating both LRP6 and *β*-catenin, and PD98059, and ERK inhibitor, can inhibit *β*-catenin accumulation [87]. Moreover, the inhibition of Wnt signaling's early stages, either through DKK1 prereceptor inhibition or Axin2 inducing *β*-catenin degradation, abolishes ERKs nuclear translocation [87], indicating the activation of ERKs and stabilization of *β*-catenin in an interdependent fashion. Inconsistent with this notion, a sustained p-ERK1/2 is required for Wnt inactivation, which is verified by the fact that U0126 (a specific ERK1/2 inhibitor) reversed Ser9 dephosphorylation and Tyr216 phosphorylation, resulting in an inhibition of GSK3*β* activation [88]. ERK1/2 inhibition facilitates GSK3*β* degradation at varied degrees, thus modulating cytoplasmic accumulation of *β*-catenin [89]. It is not clear why there is a divergence between these studies; however, it might be linked to the varying GSK3*β* compartmentalization and its effect on the signaling pathway's outcomes.

In parallel, recent studies pointed out the crosstalk between JNKs and the Wnt pathway, although the consequence is controversial. Administration of AG490, a JAK inhibitor, was documented to suppress the *β*-catenin upregulation, suggesting that JAKs act as an upstream effector of the Wnt/*β*-catenin pathway [86]. The major Drosophila Wnt homolog, wingless (Wg), has been elucidated to be activated by a JNKs pathway transcription factor activator protein-1 (AP-1). Mechanistically, essential for AP-1-driven Wg transcription is a consensus AP-1 binding site located in the second intron of Wg [90]. Of note, the Wg pathway promotes JNKs-mediated cell invasion via activating JNKs in a feedback manner, suggesting that the connections between JNKs and Wnt signaling may be a conserved mechanism in various biological processes. Alternatively, JNK2 has been demonstrated to collaborate with *β*-catenin and mediate the phosphorylation of *β*-catenin at essential residues by Rac1, which is indispensable for the activation of canonical Wnt signaling [91]. For another, JNK's phosphorylation of *β*-catenin has been observed to impede the relocation of the E-cadherin/*β*-catenin combination at cell–cell contact sites [92]. Of surprise, JNKs phosphorylates *β*-catenin on the same Ser37 and Thr41 residues as GSK3*β*, which is the cause of cytoplasmic *β*-catenin's proteasomal degradation. This leads to an intriguing supposition that the stabilization of *β*-catenin by multiple kinases may be a complex process in which cells decouple the signaling and adhesion functions of *β*-catenin. On the contrary, Hu et al. [93] demonstrated that deficiency of JNK2 limited *β*-catenin degradation in HEK293T cells. JNK2 activation led to an accumulation of GSK3*β* and a subsequent downregulation of *β*-catenin via ubiquitination and subsequent proteasomal degradation, which was almost completely abolished in GSK3*β*-inhibited or *β*-catenin-mutant groups [93]. In addition, physical interaction and colocalization were observed by immunoprecipitation assay, and results showed that active JNK2 directly interacts with GSK3*β* and *β*-catenin, suggesting that JNK2 interacts with and suppresses Wnt/*β*-catenin signaling in which GSK3*β* play a vital role. The discrepant outcomes may be attributed to the positioning of JNKs in the canonical Wnt pathway, contingent on the tissue, duration, or subcellular location of its activation.

In separate settings, increased phosphorylated P38 facilitates the Wnt signaling via enhancing the expression of *β*-catenin, and in the meantime, it down-regulated GSK3*β* expression [89]. P38 silencing negatively regulates *β*-catenin via limiting the degradation of GSK3*β*, suggesting that P38 may modulate GSK3*β* and subsequent cellular events such as the cytoplasmic accumulation of *β*-catenin [89]. In consistence, suppression of P38 using siRNAs or expression of a dominant-negative mutant of P38 is shown to restore Wnt3a-attenuated GSK3*β* kinase activity and attenuate *β*-catenin stabilization and subsequent LEF/TEF-sensitive transcription [94]. Mechanically, P38 MAPKs inactivate GSK-3*β* via direct phosphorylation at Ser^389^ comparable to the phosphorylation of Ser9 by Akt, which is the best-characterized mechanism for inhibiting GSK-3*β* activity [95]. In addition, Ehyai et al. [96] have shown that a significant decrease in *β*-catenin nuclear accumulation in Wnt3a-stimulated multiple cell types was observed when P38 MAPKs function was lost, whereas active P38 MAPKs signaling augmented *β*-catenin nuclear localization and target gene activity. Of importance, a similar positive influence on Wnt signaling modulated by P38 MAPKs has been observed upon pathogenic microbial infections. In this context, P38 MAPKs were demonstrated to be involved in the accumulation of nuclear location of *β*-catenin during *H. parasuis* infection via modulating the expression of dickkofp1(DKK1), thereby activating the role of Wnt/*β*-catenin signaling in anti-inflammation [43, 97]. In addition, blockage of DKK-1 led to the dysfunction of P38 MAPKs in modulating Wnt/*β*-catenin signaling [97], which provided a more comprehensive insight into the ability to promote the Wnt/*β*-catenin activity by P38 MAPKs may depend on the coordination of DKK1 expression. The activation of P38 MAPKs, which then caused DKK1 inhibition, was a process similar to that seen in TGF*β*-mediated fibrotic diseases, thus initiating the Wnt signaling activation [98]. However, the regulatory effect of P38 MAPKs on DKK1 expression was divergent and different in various cell types [99, 100]. One reasonable explanation is that the promoter region of the DKK1 gene may contain positive and negative regulatory elements responsible for mediating DKK1 expression with the opposite consequence [97]. Additionally, it is speculated that P38 MAPKs may trigger different regulatory elements under various conditions to switch the regulation of DKK-1 expression, and such specific mechanisms require further research. These observations above provide a mechanistic perspective into a fundamental level of crosstalk between P38 MAPKs and canonical Wnt/*β*-catenin pathway.

Taken together, in most cases, inhibition of PI3K/Akt, TLRs/NF-*κ*B, and MAPKs activation interrupts Wnt signaling to a certain extent; however, their activation does not appear to be an indispensable step in the activation of canonical Wnt/*β*-catenin pathway. It seems possible that these signals and Wnt/*β*-catenin pathways operate in a “parallel-input” scenario. At GSK3*β*, upstream of *β*-catenin accumulation, they primarily feed into the Wnt signaling, thus influencing downstream effectors that are mediated by Wnt. Nevertheless, the mechanism of how they connect, especially in response to microbial infection, is not fully understood. Consequently, the nature of signaling intermediates between various signal coordination proteins and GSK-3*β* inactivation awaits further study.

## 6. Concluding Remarks

At present, several signaling transduction pathways have been well characterized to play vital roles in the motivation and maintenance of inflammation; however, the cellular mechanisms that negatively regulate excessive inflammation are not fully illustrated. Here, we emphasized a negative effect of canonical Wnt/*β*-catenin signaling on pathogenic bacteria-induced inflammation, wherein active Wnt signaling suppresses a pro-inflammatory response and shields tissues from intense inflammation. It is well demonstrated that Wnt/*β*-catenin signaling exerts its functions via cross-talking with other inflammatory-associated pathways to precisely modulate cellular reactions. To better design interventions for treating inflammatory diseases, it is necessary to further investigate the interconnections between various inflammation-related signaling pathways in both space and time through the use of suitable cell or animal models and diverse pathogenic bacteria.

## Figures and Tables

**Table 1:** Cell-type/bacteria–stimuli-dependent regulatory effects of canonical Wnt/*β*-catenin pathway in anti-inflammation.

Regulation manners	Cell types	Stimulus (bacterium or bacterial PAMPs)	Suppression or induction of Inflammation		Reference
			Pro-inflammatory molecules	Anti-inflammatory molecules	
↑Wnt3a addition	Murine macrophages	*M. tuberculosis*	TNF↓	—	[12]
↑Wnt3a addition	Murine macrophages	*M. bovis* BCG	TNF*α*↓ IL-6↓	—	[13]
↑Wnt3a addition	Murine alveolar macrophages	*P. aeruginosa*	IL-1*β*↓ TNF*α*↓ MIP-2↓	—	[14]
↑Wnt6 addition	Murine macrophages	*M. tuberculosis*	TNF*α*↓	Arg-1↑ MRC-1↑	[16]
↓Wnt6 knockdown	Murine macrophages	*M. tuberculosis*	TNF*α*↑	Arg-1↓ MRC-1↓	[16]
↓Wnt3a Inhibition	Human monocyte cells	LPS	IL-6↑ IL-12p40↑ TNF*α*↑	—	[3]
↑Wnt activation	Human umbilical vein endothelial cells	Heat-inactivated *Rickettsia conorii*	IL-6↓ IL-8↓ CXCL1↓	—	[18]
↓Wnt inhibition	Human cancer cell lines	LPS	IL-1*β*↑	—	[19]
↑Wnt2 overexpression	Mouse intestinal epithelial cells	*Salmonella*	IL-8↓	—	[20]
↑Wnt11 overexpression	Rat small intestinal epithelial cells	*Salmonella*	IL-8↓	—	[21]
↓Wnt1 knockdown	Human colonic epithelial cells	*Salmonella*	IL-6↑ IL-8↑ GM-CSF↑	—	[26]
↓GSK3*β* inhibition	Murine macrophages	*M. avium*	TNF*α*↓	—	[12]
↓GSK3*β* inhibition	Murine peritoneal macrophages	*F. tularensis*	TNF*α*↓ IL-6↓ IL-12p40↓	IL-10↑	[27]
↓GSK3*β* knockdown	Murine peritoneal macrophages	*F. tularensis*	TNF*α*↓	IL-10↑	[27]
↓GSK3*β* inhibition	Human monocytes	LPS	TNF*α*↓ IL-6↓ IL-12p40↓	IL-10↑	[28]
↓GSK3*β* inhibition	Hamster kidney cells	Avian infectious bronchitis virus (IBV)	TNF*α*↓ IL-1*β*↓	—	[29]
↓GSK3*β* inhibition	Chicken embryonic fibroblast cells	Marek's disease virus (MDV)	IL-1*β*↓	—	[30]
↓GSK3*β* inhibition	Monkey embryonic kidney epithelial cells	Porcine reproductive and respiratory syndrome virus (PPRSV)	TNF*α*↓ IL-1*β*↓ IL-6↓ IL-8↓	—	[31]
↓GSK3*β* knockdown	Murine microglia	LPS	—	IL-10↑	[32]
↓GSK3*β* inhibition	Mouse microglia	Heat-inactivated *S. aureus*	TNF*α*↓	IL-10↑	[33]
↓GSK3*β* inhibition and knockdown	Primary murine microglia	LPS	TNF*α*↓	—	[34]
↓GSK3*β* inhibition	Colonic epithelial cells	*Salmonella typhimurium*	IL-8↓	—	[35]
↓GSK3*β* inhibition and knockdown	Primary astrocytes	LPS	IL-6↓	—	[36]
↓GSK3*β* inhibition	Primary microglia	LPS	IL-6↓	—	[36]
↑GSK3*β* overexpression	Primary astrocytes	LPS	IL-6↑	—	[36]
↑*β*-catenin overexpression	Mouse bone marrow-derived neutrophils	*P. aeruginosa*	IL-6↓ TNF*α*↓ MIP-2↓	—	[37]
↑*β*-catenin overexpression	Murine macrophages	*P. aeruginosa*	IL-1*β*↓ IL-6↓ TNF*α*↓ MIP-2↓	—	[37]
↑*β*-catenin overexpression	Human alveolar type II epithelial cells	*M. bovis* BCG or LPS	IL-1*α*↓ IL-2↓ IL-6↓ IL-8↓ TNF*α*↓	—	[40]
↓*β*-catenin knockdown	Murine macrophages	LPS	IL-6↑	—	[42]
↑*β*-catenin overexpression	Human intestinal epithelial cells	*Salmonella*	IL-8↓	—	[41]
↓*β*-catenin knockdown	human monocytes	LPS	IL-6↑ IL-12p40↑ TNF*α*↑	—	[3]
↓*β*-catenin inhibition	Chicken embryonic fibroblast cells	Marek's disease virus (MDV)	IFN*β*↑ IL-8↑	—	[30]
↓*β*-catenin knockdown	Mouse bone marrow-derived DCs	LPS	IL-6↑ IL-12p40↑ TNF*α*↑ CXCL-10↑	—	[44]
↓*β*-catenin inhibition	Porcine kidney cells and new-born pig trachea cells	*Helicobacter pylori*	IL-8↑ CCL4↑ CCL5↑	—	[43]

↑ means pro-inflammation; ↓ means anti-inflammation.
